# Long-term exposure to abnormal glucose levels alters drug metabolism pathways and insulin sensitivity in primary human hepatocytes

**DOI:** 10.1038/srep28178

**Published:** 2016-06-17

**Authors:** Matthew D. Davidson, Kimberly R. Ballinger, Salman R. Khetani

**Affiliations:** 1 School of Biomedical Engineering, Colorado State University, Fort Collins, CO 80523, USA; 2 Department of Bioengineering, University of Illinois, Chicago, IL 60607, USA; 3 Department of Mechanical Engineering, Colorado State University, Fort Collins, CO 80523, USA

## Abstract

Hyperglycemia in type 2 diabetes mellitus has been linked to non-alcoholic fatty liver disease, which can progress to inflammation, fibrosis/cirrhosis, and hepatocellular carcinoma. Understanding how chronic hyperglycemia affects primary human hepatocytes (PHHs) can facilitate the development of therapeutics for these diseases. Conversely, elucidating the effects of hypoglycemia on PHHs may provide insights into how the liver adapts to fasting, adverse diabetes drug reactions, and cancer. In contrast to declining PHH monocultures, micropatterned co-cultures (MPCCs) of PHHs and 3T3-J2 murine embryonic fibroblasts maintain insulin-sensitive glucose metabolism for several weeks. Here, we exposed MPCCs to hypo-, normo- and hyperglycemic culture media for ~3 weeks. While albumin and urea secretion were not affected by glucose level, hypoglycemic MPCCs upregulated CYP3A4 enzyme activity as compared to other glycemic states. In contrast, hyperglycemic MPCCs displayed significant hepatic lipid accumulation in the presence of insulin, while also showing decreased sensitivity to insulin-mediated inhibition of glucose output relative to a normoglycemic control. In conclusion, we show for the first time that PHHs exposed to hypo- and hyperglycemia can remain highly functional, but display increased CYP3A4 activity and selective insulin resistance, respectively. In the future, MPCCs under glycemic states can aid in novel drug discovery and mechanistic investigations.

The liver maintains blood glucose levels in a tight range (~3.9–7.2 mM glucose) by carefully balancing its own glucose output with the absorption of glucose in peripheral tissues. In type 2 diabetes mellitus (T2DM), blood glucose levels rise into the hyperglycemic range (>7.2 mM blood glucose), which is associated with many detrimental effects throughout the body^1^. In the liver itself, the prominent effect of high glucose is ectopic lipid accumulation in hepatocytes called steatosis^2^. Steatosis is the hallmark feature of non-alcoholic fatty liver disease (NAFLD), which is currently the most prevalent liver aliment and has been implicated in causing altered drug metabolism^3^ and non-alcoholic steatohepatitis (NASH)^4^. Conversely, the liver can experience hypoglycemia (<3.9 mM blood glucose) from fasting, cancer and T2DM drugs (i.e. oral hypoglycemic drugs). Fasting induces changes in cytochrome P450 (CYP450) pathways, which could alter drug efficacy and toxicity^5^,^6^. Importantly, the liver is the main site for cancer metastases, which places this tissue at risk for local glucose deprivation^7^,^8^. Furthermore, hypoglycemia is common in patients on T2DM drugs and is associated with cardiovascular disease^9^,^10^.

Owing to its critical role in glucose homeostasis, the liver is emerging as an essential target for metabolic disorder therapies^11^,^12^. Animal models of metabolic disease, such as diabetic (db/db) and high fat diet fed mice, have provided important details on disease pathogenesis^13^,^14^. However, there are significant differences in the progression of metabolic disease and drug metabolism pathways between animals and humans^15^,^16^. Thus, animal data needs to be supplemented with assays designed using *in vitro* models of the human liver, which include liver slices, cell lines and isolated primary human hepatocytes (PHHs) cultured on adsorbed or gelled extracellular matrices (ECM) such as collagen and Matrigel^17^. Liver slices are known to rapidly lose viability *in vitro* and are not amenable to high-throughput drug screening^17^. Cancerous and immortalized hepatic cell lines contain abnormal levels of drug metabolism enzymes and display an improper metabolism of energy sources^18^,^19^. On the other hand, untransformed PHHs are relatively simple to use in a medium-to-high throughput culture format for fundamental investigations and drug screening^20^. However, in the presence of ECM manipulations alone, PHHs display a precipitous decline in liver functions such as CYP450 activities, glucose metabolism and responsiveness to insulin and glucagon^21^,^22^. The aforementioned limitations with *in vitro* models of the human liver have thus hampered the investigation of how various stimuli (i.e. hypo- and hyperglycemia) affect cellular phenotypes in ways that have implications for the progression and treatment of diseases such as T2DM, NAFLD, and NASH.

Microfabrication tools can be utilized to control the extent of both homotypic interactions between PHHs and their heterotypic interactions with supportive nonparenchymal cells (NPCs), which has been shown to significantly enhance the functions and lifetime of PHHs *in vitro*^17^. Indeed, micropatterned co-cultures (MPCCs) of PHHs and 3T3-J2 murine embryonic fibroblasts can maintain prototypical hepatic morphology, secrete albumin and urea, and display high levels of CYP450 activities for 4–6 weeks^21^,^23^. In this model, PHHs are first organized onto collagen-coated circular domains of empirically optimized dimensions in 24- or 96-well plates using semiconductor-driven soft-lithographic tools and then surrounded by 3T3-J2 fibroblasts. More recently, we have shown that MPCCs created using cryopreserved PHHs maintain glucose metabolism and sensitivity to insulin and glucagon for several weeks^22^. In particular, MPCCs retained the ability to store glycogen and produce glucose (via gluconeogenesis or glycogen lysis) under the nutritional states of feeding and fasting, respectively. Furthermore, such glucose metabolism in MPCCs responded robustly to insulin and glucagon with *in vivo*-like trends.

In this study, we utilized MPCCs to investigate for the first time the effects of chronic stimulation (~3 weeks) with pathophysiologic glucose levels (hypo- and hyperglycemia) on several aspects of the PHH phenotype, which included: hepatocyte morphology, gene expression, albumin secretion, urea synthesis, CYP450 activities, hepatic lipid accumulation, and the effects of insulin on glucose output. We compared our results to a normoglycemic control condition.

## Results

PHHs in MPCCs were initially stabilized in a normoglycemic culture medium (5 mM glucose) for 4 days to enable fibroblasts to reach a confluent monolayer across all conditions, and then exposed for up to 18 days to culture media supplemented with different glucose levels. In order to induce a hypoglycemic state, no additional glucose was added to the culture medium except for the amount of glucose (0.4–0.5 mM) that was present in the 10% vol/vol bovine serum used. In order to mimic a hyperglycemic state, glucose was added to the serum-supplemented culture medium to yield a concentration of 25 mM, which was also chosen as a comparator to the high glucose culture media typically utilized for hepatocyte culture^24^.

**Figure 1:**
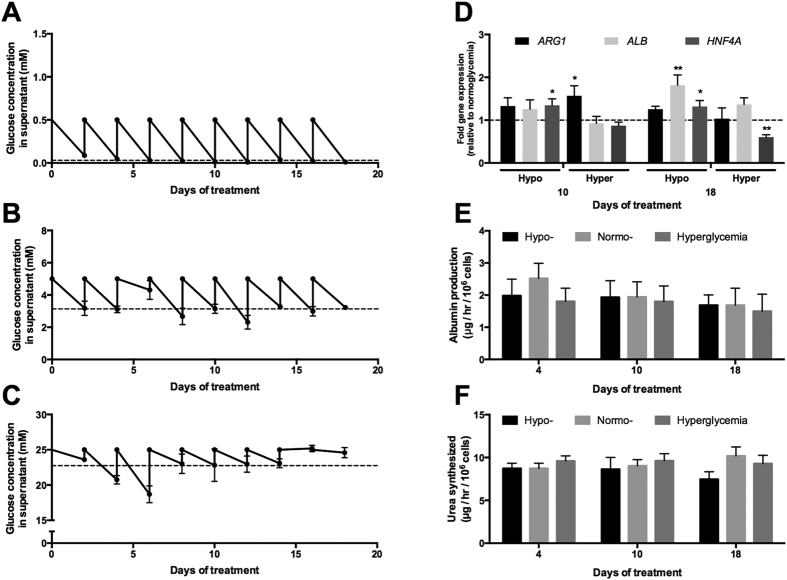
Albumin and urea secretion in MPCCs treated with varying levels of glucose. Fluctuations in culture medium glucose concentration in hypo- (**A**), normo- (**B**) and hyperglycemic (**C**) MPCCs over 3 weeks of culture. All cultures were initially cultured in a normoglycemic (5 mM glucose) culture medium for 4 days and then switched to their respective glycemic conditions (0.4–0.5 mM glucose for hypoglycemic and 25 mM glucose for hyperglycemic). Glucose levels in the depleted culture medium were measured with every 2-day culture media exchange. Dashed lines indicate average depleted glucose levels. Levels of albumin (*ALB*), arginase 1 (*ARG1*) and hepatocyte nuclear factor 4 α (*HNF4A*) mRNA transcripts (**D**) in MPCCs treated with hypo- and hyperglycemic culture media for either 10 or 18 days. Data is normalized to a normoglycemic control. Albumin (**E**) and urea secretion (**F**) in MPCCs incubated for up to 18 days in the varying glucose levels. Error bars represent SD. *p ≤ 0.05 and **p ≤ 0.01. Trends were observed in multiple hepatocyte donors.

The glucose concentrations in the spent culture supernatants were measured every 2 days at the fresh medium exchange. MPCCs in the hypo- and normoglycemic culture media consistently consumed glucose over 3 weeks in culture with average glucose fluctuations from ~0.5 mM down to 0.03 mM (Fig. 1A) and ~5 mM down to 3.11 mM (Fig. 1B), respectively. On the other hand, hyperglycemic cultures showed more erratic glucose consumption over three weeks with minor fluctuations in glucose levels at terminal time points and an average drop from ~25 mM down to 22.74 mM (Fig. 1C). The use of propidium iodide (stains nuclei of dead cells with leaky membranes) coupled with Hoechst 33342 (stains nuclei of all cells) showed no significant differences in PHH or 3T3-J2 viability in MPCCs treated with the various glucose concentrations for ~3 weeks (data not shown). Furthermore, 3T3-J2 fibroblast numbers, as measured by counting DAPI (4′,6-diamindino-2-phenylindole)-stained fibroblast nuclei after 3 weeks of culture in MPCCs, were not significantly altered in the 3 different glucose concentrations (Fig. S1). The 3T3-J2 fibroblasts are required for maintenance of PHH functions in MPCCs^21^,^23^. However, these non-liver and murine fibroblasts do not express the *human* mRNA transcripts measured here nor do they display liver functions, such as albumin secretion, urea synthesis and CYP450 activities^21^.

### Albumin and urea in MPCCs is not affected by the glycemic state

At the gene expression level, *ARG1* (arginase 1, urea cycle enzyme) was not significantly affected in hypoglycemic MPCCs, and only *transiently* upregulated in hyperglycemic MPCCs (~1.6 fold after 10 days of exposure) relative to the normoglycemic control (Fig. 1D). *ALB* (albumin) in hypoglycemic MPCCs was upregulated ~1.8 fold after 18 days of exposure relative to the normoglycemic control, while it was not affected to any significant degree in hyperglycemic MPCCs. *HNF4A* (hepatocyte nuclear factor 4 alpha) was upregulated ~1.3 fold in MPCCs after 10–18 days of exposure to a hypoglycemic culture medium, while it showed a downregulation (by ~40%) in hyperglycemic MPCCs after 18 days of exposure when compared to the normoglycemic control.

In spite of the aforementioned gene expression changes, neither the secretion of albumin (Fig. 1E) nor urea by MPCCs (Fig. 1F) was affected significantly in any of the glycemic states. These markers are used to monitor the overall ‘health’ and phenotypic stability of hepatocytes *in vitro*^24^. On the other hand, the removal of serum from the culture medium caused PHH functions to decline irrespective of glucose levels (Fig. S2).

### CYP450 pathways are modulated by glycemic states

At the gene expression level, *CYP1A2*, *CYP2B6*, *CYP2C19* and *CYP3A4* expression were significantly increased by ~2.7 fold, 3.1 fold, 1.8 fold, and 3.7 fold after 10 days of treatment with a hypoglycemic culture medium, respectively, as compared to a normoglycemic control (Fig. S3). Similar trends were observed after 18 days of treatment with a hypoglycemic culture medium such that *CYP1A2, CYP2D6*, *CYP2E1* and *CYP3A4* were increased by ~1.6 fold, 1.3 fold, 1.5 fold and 1.3 fold, respectively, relative to normoglycemic MPCCs. *CYP2A6* expression in hypoglycemic MPCCs remained within the range of the normoglycemic control.

In hyperglycemic MPCCs, we measured a ~1.5 fold increase in *CYP2A6* transcripts, while the other CYP450 transcripts (*CYP1A2, CYP2B6, CYP2C19, CYP2D6, CYP2E1*, and *CYP3A4)* were not significantly affected after 10 days of treatment relative to the normoglycemic control (Fig. S3). However, after 18 days of treatment, hyperglycemic cultures showed upregulation of *CYP1A2* (~1.3 fold) and *CYP2D6* (~1.4 fold), while *CYP2E1* was downregulated (by ~43%) as compared to the normoglycemic cultures.

**Figure 2:**
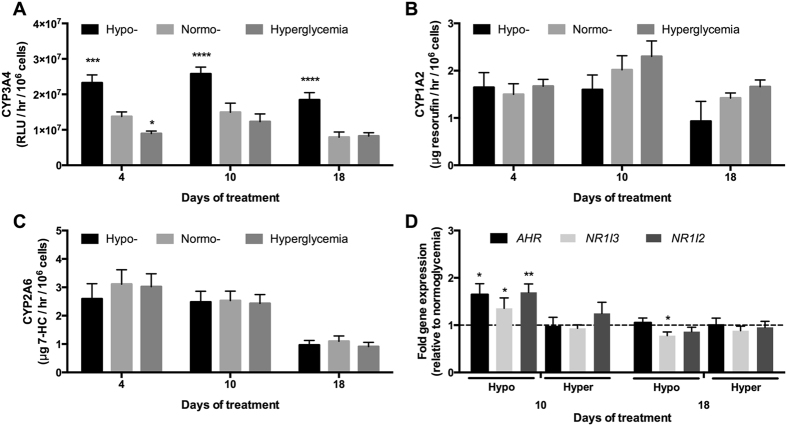
Glucose-induced modulation of CYP450 pathways in MPCCs. (**A)** CYP3A4 enzyme activity in MPCCs (as measured by cleavage of luciferin-IPA into luciferin) treated with hypo-, normo- and hyperglycemic culture media for up to 18 days. (**B**) CYP1A2 activity (as measured by cleavage of ethoxyresorufin into resorufin) in MPCCs treated as in panel (**A**). (**C**) CYP2A6 activity (as measured by conversion of coumarin into 7-hydroxycoumarin, abbreviated as 7-HC) in MPCCs treated as in panel (**A**). (**D**) Levels of aryl hydrocarbon receptor (*AHR*), constitutive androstane receptor (*NR1I3*) and pregnane X receptor (*NR1I2*) mRNA transcripts in MPCCs treated with hypo- and hyperglycemic culture media for 10 or 18 days. Data is normalized to a normoglycemic control. Error bars represent SD. *p ≤ 0.05, **p ≤ 0.001, ***p ≤ 0.01, and ****p ≤ 0.0001. Trends were observed in multiple hepatocyte donors.

When we measured CYP450 enzyme activities in MPCCs using prototypical substrates, we observed a ~1.7 fold increase in CYP3A4 activity after 4 and 10 days of treatment, and ~2.3 fold increase after 18 days of treatment with a hypoglycemic culture medium relative to the normoglycemic control (Fig. 2A). CYP3A4 activity was decreased (by ~40%) in hyperglycemic cultures after 4 days of treatment, but such a decrease was diminished after 10 and 18 days of treatment when compared to the normoglycemic cultures. On the other hand, neither CYP1A2 activity (Fig. 2B) nor CYP2A6 activity (Fig. 2C) in MPCCs was significantly affected as a function of glucose levels in the culture medium over 18 days of exposure.

The aforementioned increase in CYP3A4 activity in hypoglycemic MPCCs relative to the normoglycemic control could potentially be due to the up-regulation of *NR1I2* (nuclear receptor subfamily 1, group I, member 2, also known as pregnane X receptor or *PXR)* and *NR1I3* (nuclear receptor subfamily 1, group I, member 3, also known as constitutive androstane receptor or *CAR*) transcripts^25^ by ~1.7 fold and ~1.4 fold, respectively, after 10 days of exposure (Fig. 2D). Interestingly, the up-regulation of *AHR* (aryl hydrocarbon receptor) transcripts measured in hypoglycemic MPCCs by ~1.6 fold relative to the normoglycemic control after 10 days of exposure did not correlate with the lack of modulation observed in CYP1A2 enzyme activity^26^ (Fig. 2B). However, such upregulation of nuclear receptors at the gene expression level in MPCCs was diminished after 18 days of exposure to a hypoglycemic culture medium. None of the aforementioned nuclear receptors’ transcripts were modulated to any significant degree in hyperglycemic MPCCs as compared to the normoglycemic control.

### Hepatic lipid accumulation in MPCCs increases under hyperglycemia

**Figure 3:**
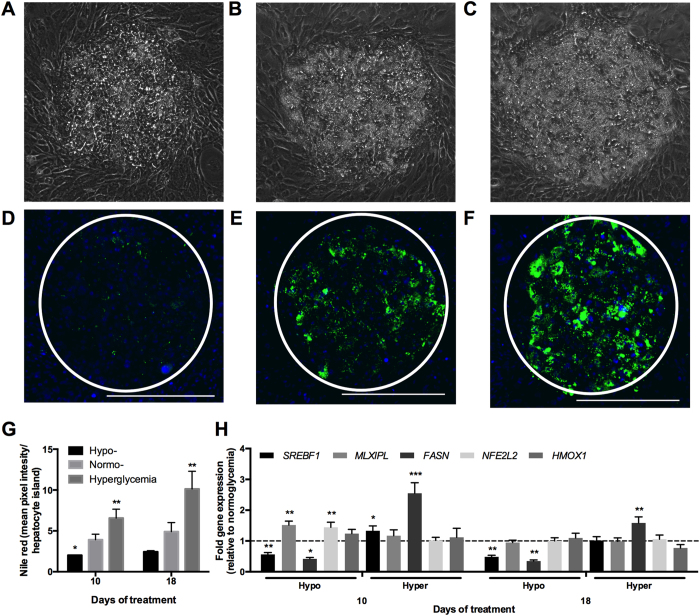
Glucose-induced accumulation of neutral lipids in MPCCs. Phase contrast images of MPCCs treated for 18 days with a hypo- (**A**), normo- (**B**) and hyperglycemic (**C**) culture medium. Nile red (neutral lipids) staining of MPCCs treated for 18 days with a hypo- (**D**), normo- (**E**) and hyperglycemic (**F**) culture medium. Scale bars represent 400 μm. (**G**) Quantification of Nile Red staining in hepatocyte islands in MPCCs subjected to hypo-, normo- or hyperglycemic culture media for 10 or 18 days. (**H**) Levels of sterol regulatory element binding protein (*SREBF1*), carbohydrate response element binding protein (*MLXIPL*), fatty acid synthase (*FASN*), nuclear factor (erythroid-derived 2)-like 2 (*NFE2L2)* and heme oxygenase 1 (*HMOX1*) mRNA transcripts in MPCCs treated with hypo- and hyperglycemic culture media for 10 or 18 days. Data is normalized to a normoglycemic control. Error bars represent SD. *p ≤ 0.05, **p ≤ 0.01, and ***p ≤ 0.001. Trends were observed in multiple hepatocyte donors.

Under all glucose treatments, PHHs cultured in MPCCs remained attached to the distinct micropatterned collagen islands and maintained prototypical *in vivo*-like morphology (i.e. polygonal shape, distinct nuclei/nucleoli, visible bile canaliculi) over ~3 weeks of treatment (Fig. S4). Additionally, we did not observe any overt changes in the morphology and spreading of fibroblasts in MPCCs as a function of glucose concentrations. However, PHHs exposed to a hyperglycemic culture medium in the presence of insulin accumulated vesicles in their cytoplasm at a greater rate/level than PHHs exposed to hypo- and normoglycemic media (Fig. 3A–C, Fig. S5A–C). The vesicles that accumulated in the hepatic cytoplasm were found to be neutral lipids (i.e. triacylglycerol and cholesterol esters) via Nile red staining (Fig. 3D–F, Fig. S5D–F). Some lipid accumulation was also observed in PHHs exposed to a normoglycemic culture medium, while PHHs exposed to a hypoglycemic culture medium accumulated little or no lipids throughout the culture duration even in the presence of insulin. Quantification of the Nile Red fluorescence intensities revealed that there were ~1.7 and ~2.1 fold more lipids after 10 days and 18 days of exposure to a hyperglycemic culture medium, respectively, when compared to a normoglycemic control (Fig. 3G). In contrast, hypoglycemic MPCCs had ~50% of the hepatic lipids found in normoglycemic MPCCs.

Consistent with the aforementioned lipid profile, *FASN* (fatty acid synthase) and *SREBF1* (sterol regulatory element binding transcription factor 1) mRNA transcripts were upregulated ~2.5 fold and ~1.3 fold, respectively, in hyperglycemic MPCCs relative to a normoglycemic control after 10 days of exposure (Fig. 3H). After 18 days of exposure, only *FASN* remained upregulated by ~1.6 fold in hyperglycemic MPCCs relative to normoglycemic cultures. In contrast, expression levels of *MLXIPL* (MLX-interacting protein-like, also known as carbohydrate responsive element binding protein or ChREBP), *HMOX1* (heme oxygenase 1, an oxidative stress marker), and *NFE2L2* (nuclear factor (erythroid-derived 2)-like 2, an oxidative stress marker) were not affected to any significant degree in hyperglycemic MPCCs when compared to a normoglycemic control.

In hypoglycemic MPCCs, *FASN* and *SREBF1* were downregulated by ~58% and ~44% after 10 days of exposure, and by ~65% and ~51% after 18 days of exposure, respectively, as compared to normoglycemic levels (Fig. 3H). Surprisingly, *MLXIPL* expression in hypoglycemic MPCCs was increased by ~1.5 fold after 10 days of exposure, although this diminished after 18 days of exposure. *HMOX1* was not increased significantly in hypoglycemic MPCCs relative to the normoglycemic control. Finally, *NFE2L2* was increased by ~1.4 fold in hypoglycemic MPCCs relative to the normoglycemic control after 10 days of exposure; however, such an increase diminished after 18 days of exposure.

### Hyperglycemic MPCCs become less sensitive to insulin’s effects on glucose output

**Figure 4:**
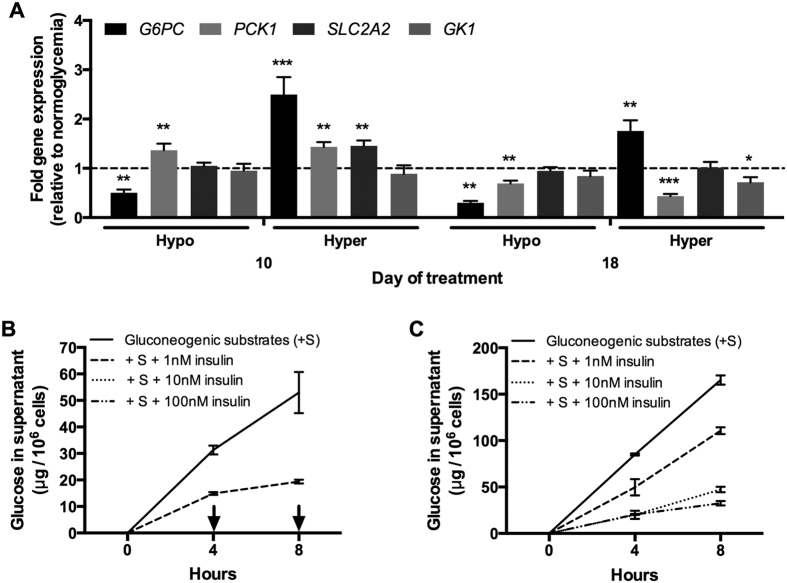
Glucose-induced modulation of glucose metabolism pathways and insulin sensitivity in MPCCs. (**A**) Levels of glucose-6-phosphatase catalytic subunit (*G6PC*), phosphoenolpyruvate carboxykinase-1 cytosolic (*PCK1*), glucose transporter 2 (*SLC2A2*) and glycerol kinase (*GK1*) mRNA transcripts in MPCCs treated with hypo- and hyperglycemic culture media for 10 or 18 days. Data is normalized to a normoglycemic control. (**B**) Glucose output in supernatants of normoglycemic MPCCs (10 days of treatment) treated with gluconeogenic substrates (20 mM lactate and 2 mM pyruvate) in the presence or absence of different levels of insulin (see methods for additional details). Arrows indicate no detectable glucose in supernatants. (**C**) Glucose output as in panel (**B**) except hyperglycemic MPCCs were used. Error bars represent SD. *p≤ 0.05, **p ≤ 0.01, and ***p ≤ 0.001. Similar results were observed in another primary hepatocyte donor.

We first probed the expression levels of genes involved in glucose metabolism in MPCCs treated with varying glucose concentrations in the culture medium. *PCK1* (phosphoenolpyruvate carboxykinase 1) gene expression was upregulated by ~1.4 fold in hypoglycemic cultures as compared to the normoglycemic control after 10 days of exposure (Fig. 4A). However, after 18 days of exposure, *PCK1* expression was downregulated in hypoglycemic cultures by ~31% of normoglycemic transcript levels. *SLC2A2* (solute carrier family 2, facilitated glucose transporter member 2) gene expression was similar between hypo- and normoglycemic cultures. On the other hand, *G6PC* (glucose-6-phosphatase, catalytic subunit) expression was downregulated in hypoglycemic cultures by ~50% and ~70% of normoglycemic levels after 10 and 18 days of exposure, respectively. Finally, *GK1* (glycerol kinase) expression was not significantly altered in hypoglycemic MPCCs as compared to normoglycemic MPCCs.

In hyperglycemic MPCCs, *PCK1* gene expression was upregulated by ~1.4 fold relative to normoglycemic controls after 10 days of exposure (Fig. 4A). However, after 18 days of exposure, *PCK1* expression was downregulated in hyperglycemic cultures by ~57% of normoglycemic transcript levels. *SLC2A2* gene expression was upregulated by ~1.5 fold in hyperglycemic cultures relative to the normoglycemic control after 10 days of exposure. Following 18 days of exposure, *SLC2A2* expression became similar across normo- and hyperglycemic MPCCs. In contrast to hypoglycemic MPCCs, *G6PC* gene expression was upregulated in hyperglycemic cultures at 10 and 18 days of exposure to ~2.5 fold and ~1.8 fold of the normoglycemic control, respectively. Lastly, *GK1* expression showed a slight downregulation in hyperglycemic MPCCs by ~28% of normoglycemic controls, but only after 18 days of exposure.

Next, we assessed the effects of insulin doses on glucose secretion from MPCCs treated with varying glucose levels. In particular, normo- and hyperglycemic MPCCs exposed to the respective glucose levels for 10 days were subsequently incubated for 1 day in hormone-free (but 5 mM glucose-supplemented) culture medium, and then placed in glucose-free and serum-free culture medium containing substrates for gluconeogenesis (20 mM L-lactate and 2 mM pyruvate) in the presence or absence of different doses of recombinant human insulin. After 8 hours of stimulation, normoglycemic hepatocytes produced ~53 μg of glucose per million hepatocytes in supernatants (Fig. 4B), while hyperglycemic hepatocytes produced 165 μg of glucose per million hepatocytes (Fig. 4C). When incubated with a 1 nM dose of insulin, glucose output from normoglycemic and hyperglycemic MPCCs was reduced by ~64% and ~33% of insulin-free controls, respectively. Increasing the insulin doses to 10 and 100 nM completely inhibited glucose output from normoglycemic MPCCs. On the other hand, glucose output was still detected at high levels (32–47 μg/million hepatocytes) in supernatants from hyperglycemic MPCCs treated with 10 and 100 nM insulin, suggesting an insulin resistant phenotype in hyperglycemic MPCCs relative to the normoglycemic control.

A time-course assessment showed that the aforementioned insulin resistance trends started appearing in hyperglycemic MPCCs after 6 days of exposure (but not after 2 days) and persisted through the exposure period of 18 days (Fig. S6). Furthermore, reducing the glucose concentration in a hyperglycemic culture medium down to 12.5 mM led to a similar amount of lipid accumulation as observed in the 25 mM glucose concentration. However, significant insulin resistance was only observed in the 25 mM glucose culture medium when compared to the normoglycemic control. Lastly, insulin resistance was also observed in hyperglycemic MPCCs relative to the normoglycemic control when the PHHs were allowed to produce glucose in supernatants in the *absence* of gluconeogenic substrates (Fig. S7). Without supplemented gluconeogenic substrates, PHHs can presumably produce glucose from endogenous sources (i.e. free amino acids and glycerol) and/or the lysis of built-up glycogen stores, which were found to be similar across the normo- and hyperglycemic MPCCs *prior* to the glucose output assay described above (Fig. S7).

**Figure 5:**
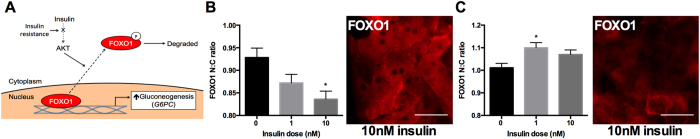
Insulin-induced translocation of FOXO1 in hepatocytes. (**A**) Schematic illustrating the effect of insulin on translocation of transcription factor, FOXO1 (forkhead box O1) from the nucleus to the cytoplasm. FOXO1 typically stimulates gluconeogenic gene expression in the nucleus; however, in the presence of insulin, Akt phosphorylates FOXO1, thereby causing its translocation from the nucleus to the cytoplasm where it is degraded. MPCCs treated with a hyperglycemic or normoglycemic culture medium for 10 days were treated for 1 hour with 0, 1 or 10 nM insulin and then FOXO1 protein localization was visualized via immunostaining (see methods for additional details). (**B**) Quantification of nuclear to cytoplasmic (N:C) ratio of FOXO1 labeling in primary human hepatocytes within normoglycemic MPCCs after stimulation with or without insulin doses (n = 205 cells per treatment). Representative image of the FOXO1 labeling is shown to the right. (**C**) Quantification of N:C ratio of FOXO1 and representative image of the FOXO1 labeling in hyperglycemic MPCCs (n = 205 cells per treatment). Scale bars are 30 μm. Error bars represent SD. *p ≤ 0.05, **p ≤ 0.01, and ***p ≤ 0.001.

As an alternative measure of altered insulin signaling in hyperglycemic MPCCs relative to their normoglycemic counterparts, we assessed FOXO1 (forkhead box O1) levels in the nucleus and cytoplasm of cultures after stimulation with different doses of insulin for 1 hour. FOXO1 is a transcription factor involved in the transcription of gluconeogenic genes. Normal insulin signaling leads to the translocation of FOXO1 from the nucleus to the cytoplasm where it is degraded, thereby decreasing gluconeogenesis^27^ (Fig. 5A). The nuclear to cytoplasmic ratio (N:C) of FOXO1 has been previously used to assess FOXO1 sensitivity to insulin stimulation^28^. Here, we found that normoglycemic PHHs in MPCCs showed a reduction in the FOXO1 N:C ratio from 0.93 in an insulin-free control down to 0.87 and 0.84 with a 1 nM and 10 nM insulin stimulation, respectively (Fig. 5B). On the other hand, hyperglycemic PHHs in MPCCs showed an increase in the FOXO1 N:C ratio from 1.01 in an insulin-fee control up to 1.1 and 1.07 in 1 nM and 10 nM insulin simulation, respectively (Fig. 5C).

## Discussion

Hyperglycemia in T2DM has been implicated in the development of NAFLD, which can further exacerbate insulin resistance and progress to NASH, fibrosis/cirrhosis and hepatocellular carcinoma^29^. Conversely, hypoglycemia in the liver due to fasting, cancer or the effects of T2DM drugs can induce changes in drug metabolism pathways, which can potentially alter drug efficacy and/or toxicity^5^,^6^,^7^,^8^. *In vitro* models of the human liver can supplement animal studies for investigating the progression of disease profiles and drug outcomes given species-specific differences in drug metabolism^15^,^24^. PHHs are considered to be the ‘gold standard’ for creation of such models; however, these cells rapidly decline in their phenotypic functions in conventional monolayers, including sensitivity to insulin and glucagon^22^. Such a functional decline can be mitigated for a few weeks when PHHs are placed in MPCCs^22^. Here, we incubated MPCCs in a hypo- or hyperglycemic culture medium for ~3 weeks and compared phenotypic changes to those observed in a normoglycemic control. We observed for the first time that PHHs in MPCCs: a) maintain albumin and urea secretions at similar levels across all glycemic states; b) significantly upregulate CYP3A4 activity under hypoglycemia; and, c) accumulate neutral lipids while becoming less sensitive to insulin-mediated reduction in glucose output under hyperglycemia as compared to a normoglycemic control.

Surprisingly, we found that besides what was found in serum (~0.4–0.5 mM glucose in medium containing 10% vol/vol serum), no additional glucose supplementation in the culture medium was required to enable the survival of 3T3-J2 fibroblasts as well as the survival and major functions (i.e. albumin and urea secretion) of PHHs in such hypoglycemic MPCCs after ~3 weeks of treatment as compared to other glycemic states. Additionally, oxidative stress markers (*HMOX1* and *NFE2L2* gene expression) did not vary significantly across MPCCs treated with varying glucose levels, except for a transient upregulation in hypoglycemic MPCCs (~1.4 fold relative to a normoglycemic control). However, removal of serum from the culture medium caused PHH functions to decline irrespective of glucose concentrations, which is likely due to the lack of fibroblast spreading and growth in serum-free medium, along with other unidentified factors in serum that may be beneficial for PHHs. Thus, our results suggest that the levels of glucose (10–25 mM) present in many culture media bases (i.e. Williams E, DMEM, Alpha-MEM)^21^,^30^ might not be necessary for high functions of *stable* PHHs in co-culture with stromal cells; however, serum is a required supplement.

While albumin and urea secretion were not affected in hypoglycemic MPCCs, expression of CYP450 transcripts (*1A2, 2B6, 3A4, 2C19*) consistently showed significant upregulation relative to a normoglycemic control. Such increases in CYP450 gene expression are likely due to the measured upregulation of NR gene expression (*AHR, PXR, CAR)* in hypoglycemic MPCCs. Specifically, PXR and CAR activation induce hepatic CYP3A4 expression^25^, while AHR activation induces CYP1A2 expression^26^. NRs are also sensitive to cellular energy status and can thus mediate glucose metabolism^31^. At the enzyme activity level, CYP1A2 and CYP2A6 were not affected under hypoglycemia as compared to normoglycemia; however, hypoglycemia led to a significant (up to 2.3 fold) increase in CYP3A4 activity, an enzyme involved in the metabolism of >50% of drugs on the market^32^. Such an upregulation in CYP3A4 activity could have clinical implications for altered drug efficacy and/or toxicity. While our results in PHHs over long-term culture are novel, they are consistent with the activation of PXR and upregulation of CYP3A11 (ortholog of CYP3A4) during fasting of rodents^5^,^6^,^33^.

As with hypoglycemia, hyperglycemia did not affect albumin and urea secretion as compared to a normoglycemic control. However, expression of some CYP450 transcripts (*2A6, 1A2*, *2D6)* showed a slight (~1.3–1.5 fold) upregulation, while *2E1* was downregulated when MPCCs were exposed to a hyperglycemic culture medium as compared to a normoglycemic control. In spite of the aforementioned gene expression changes, CYP1A2 and CYP2A6 activities were not affected in hyperglycemic MPCCs relative to the other glycemic states. Consistent with its gene expression, CYP3A4 activity was similar between hyperglycemic and normoglycemic MPCCs. The lack of changes in measured CYP450 activities in hyperglycemic MPCCs are not always consistent with changes in CYP450 activities observed in patients with NAFLD, such as decreased CYP3A4/3A5 activity^34^,^35^,^36^, decreased CYP1A2 activity^35^,^37^,^38^ and increased CYP2A6 activity^38^. However, statistical confidence is not always reached in clinical studies due to significant patient-to-patient variabilty^38^. While our CYP450 results could be due to variability in response across PHH donors, they could also suggest that hyperglycemia and ensuing lipid accumulation are not responsible for all the changes in drug metabolism enzymes seen in NAFLD patients. In addition to any lipid accumulation due to hyperglycemia, fatty acids delivered to the liver from adipose tissue also contribute to NAFLD^39^. Therefore, the effects of different types of fatty acids on long-term drug metabolism changes in PHHs merits further investigation.

Similar to T2DM patients, a hyperglycemic state induced between 1.7 fold and 2.1 fold greater accumulation of neutral lipids (i.e. triacylglycerol and cholesterol esters) in PHHs relative to a normoglycemic control. Furthermore, hyperglycemic cultures had elevated *FASN* and *SREBF1* gene expression, whereas the expression of *MLXIPL* was unchanged by hyperglycemia as compared to normoglycemic cultures. The *SREBF*1 gene encodes for SREBP-1c, an insulin-responsive transcription factor that regulates *de novo* lipogenesis, including the expression of *FASN*^40^. Thus, it is likely that the increased lipid accumulation observed in hyperglycemic MPCCs is due to the excess glucose being shuttled into insulin-stimulated lipogenesis pathways^41^. On the other hand, hypoglycemic MPCCs showed significant downregulation (~50%) of *FASN* and *SREPF1* gene expression and neutral lipid accumulation relative to normoglycemic MPCCs.

Hyperglycemic MPCCs became less sensitive to insulin’s effects on gluconeogenesis over time relative to the normoglycemic control (i.e. insulin resistance). Such a trend was also observed in the absence of supplemented gluconeogenic substrates. The increase in glucose output from hyperglycemic MPCCs correlated with significantly higher expression of *G6PC* as compared to the normoglycemic control. Interestingly, *HMOX1*, a positive predictor of insulin resistance in obese individuals^42^, was not altered in hyperglycemic PHHs at the gene expression level. While we cannot rule out differential protein activity, the lack of changes in the levels of transcripts potentially suggest a different mechanism by which hyperglycemia induces insulin resistance in PHHs relative to the insulin resistance observed in individuals whose livers are being affected by both *de novo* lipogenesis and the delivery of fatty acids from adipocytes. Importantly, we found that FOXO1 displacement from the nucleus following stimulation with insulin, which normally inhibits gluconeogenesis, was significantly decreased in hyperglycemic PHHs relative to the normoglycemic PHHs. Since insulin-stimulated pathways, namely AKT-mediated phosphorylation, cause the translocation of FOXO1 from the nucleus to the cytoplasm^43^, it is conceivable that this arm of the insulin signaling pathway is inhibited under hyperglycemia in MPCCs. However, further cell signaling studies would be required to probe this mechanism.

To our knowledge, our study constitutes the first time that ‘selective’ insulin resistance has been observed in hyperglycemic PHHs such that insulin continued to simulate lipid accumulation (steatosis) but did not reduce glucose output to the same extent as the normoglycemic control. Complete loss of insulin signaling (i.e. lipogenesis and glucose production) can be achieved using liver-specific insulin receptor knockout mice^44^. However, the selective insulin resistance we observed in PHHs is more akin to a T2DM state in humans in which insulin fails to suppress gluconeogenesis but continues to activate lipogenesis, thereby leading to hyperglycemia, hypertriglyceridemia and steatosis^45^. Hypertriglyceridemia has been linked to lipotoxicity in multiple organs, including NASH^46^. Furthermore, the resulting hyperglycemia due to selective insulin resistance continues to stimulate insulin production from the pancreas, which causes the classic triad of hyperinsulinemia, hyperglycemia, and hypertriglyceridemia in patients with T2DM. We plan to evaluate the effects of long-term exposure to these three stimuli on MPCC functions in follow-up studies.

While our results provide fundamental insights into how varying glucose levels affect the PHH phenotype, the liver also contains NPCs, such as Kupffer macrophages (KMs), sinusoidal endothelial cells and stellate cells, that can modulate NAFLD/NASH progression^47^,^48^. The MPCC platform is ‘modular’ in that controlled interactions between PHHs and different NPCs can be studied without significant changes to PHH homotypic interactions on the micropatterned ECM domains. In our experience, 3T3-J2 fibroblasts induce higher levels of functions in PHHs than liver NPCs (manuscript in preparation). However, liver NPCs can be cultured within or on top of the fibroblast layer to provide a better physiological context while retaining high hepatic functions. For instance, KMs can be cultured atop pre-established MPCCs to study the effects of KM activation on hepatic CYP450s^49^. This model can also be useful to study how PHHs and KMs interact when cultured in a NAFLD/NASH-like milieu (i.e. hyperglycemia). Other groups are pursuing inclusion of stellate cells into PHH models^50^, which could be useful to understand the role of these cells in liver diseases.

In conclusion, we show the utility of long-term PHH cultures for understanding how energy sources lead to changes in glucose metabolism and hormonal responsiveness that have been implicated in T2DM, NAFLD and NASH. In the future, MPCCs could be useful for novel drug discovery efforts for such diseases. Ultimately, coupling MPCCs with other tissue types on a microfluidic chip can allow a systems level exploration of disease progression.

## Materials and Methods

### Cell culture

Cryopreserved primary human hepatocytes (PHHs) were purchased from vendors permitted to sell products derived from human organs procured in the United States of America by federally designated Organ Procurement Organizations (BioreclamationIVT, Baltimore, MD; Triangle Research Laboratories, Durham, NC). Work with human cells was conducted at Colorado State University and University of Illinois at Chicago with the approval of the Institutional Biosafety Committees at each institution. PHHs were thawed, counted and viability was assessed as previously described^23^. Micropatterned co-cultures (MPCCs) were created as previously described^21^. Briefly, adsorbed rat tail collagen I (Corning Life Sciences, Tewksbury, MA) was lithographically patterned in each well of a 24-well or 96-well plate to create 500 μm diameter circular domains spaced 1200 μm apart, center-to-center. PHHs selectively attached to the collagen domains leaving ~30,000 attached PHHs on ~85 collagen-coated islands within each well of a 24-well plate or ~4,500 attached PHHs on ~13 collagen-coated islands within each well of a 96-well plate. 3T3-J2 murine embryonic fibroblasts were seeded 18 to 24 hours later at ~90,000 cells per well in a 24-well plate or ~15,000 cells per well in a 96-well plate to create MPCCs. Culture medium containing ~5 mM D-glucose (Fisher BioReagents, Pittsburgh, PA) in a DMEM base (Dulbecco’s Modified Eagle’s Medium, Corning Life Sciences) was replaced on MPCCs every 2 days (300 μL/well for 24-well plate and 50 μL/well for 96-well plate). Other components of the MPCC culture medium have been described previously^51^. Four days after establishment of the contact-inhibited fibroblast monolayer adjacent to the confluent PHH clusters, MPCCs were treated with varying D-glucose levels, including hypoglycemia (~0.4–0.5 mM), normoglycemia (~5 mM) and hyperglycemia (~25 mM). For the hypoglycemic culture medium, no additional glucose was supplemented in the culture medium except for what was present in the 10% vol/vol serum used.

### Gene expression profiling

Total RNA was isolated and purified using the RNeasy mini kit (Qiagen, Valencia, CA) and genomic DNA was digested using the Optizyme recombinant DNase-I digestion kit (Fisher BioReagents). Approximately 10 μL of purified RNA was reverse transcribed into complementary DNA (cDNA) using the high capacity cDNA reverse transcription kit (Applied Biosystems, Foster City, CA). Then, 250 ng of cDNA was added to each qPCR (quantitative polymerase chain reaction) reaction along with the Solaris™ master mix and pre-designed human-specific primer/probe sets according to manufacturer’s protocol (GE Healthcare Dharmacon, Lafayette, CO). The primer and probe sequences provided by GE Healthcare Dharmacon are provided in Supplemental Table 1. Additionally, for some genes that included: *SREBF1* (ID: HS01088691_m1), *FASN* (ID: HS01005622_m1), *GK1* (ID: HS04235340_s1), *NFE2L2* (ID: HS00975961_g1)*, CYP1A2* (ID: HS00167927_m1), *CYP2C19* (ID: HS00426380_m1)*, CYP2E1* (ID: HS00559368_m1)*, CYP2B6* (ID: HS03044636_m1)*, CYP2D6* (ID: HS00164385_m1)*, HNF4A* (ID: HS00230853_m1)*, NR1I2* (ID: HS01114267_m1) and *NR1I3* (ID: HS00901571_m1), Taqman™ (Thermo Fisher Scientific, Waltham, MA) master mix and primer/probe sets were used according to manufacturer’s protocol. The Taqman primer/probe sets were selected to be human-specific without cross-reactivity to mouse DNA; however, primer/probe sequences are proprietary to the manufacturer. The primer/probe sets were also tested with pure mouse 3T3-J2 fibroblast cDNA and found to not cross-react. qPCR was performed on a Mastercycler Realplex instrument (Eppendorf, Hamburg, Germany), and data were analyzed using the comparative C(T) method. Gene expression was normalized to the housekeeping gene, glyceraldehyde 3-phosphate dehydrogenase (*GAPDH*), the expression of which did not vary in MPCCs as a function of glucose levels over at least 18 days of exposure (data not shown).

### Biochemical assays

Urea concentration in collected cell culture supernatants was assayed using a colorimetric endpoint assay utilizing diacetyl monoxime with acid and heat (Stanbio Labs, Boerne, TX). Albumin levels were measured using an enzyme-linked immunosorbent assay (MP Biomedicals, Irvine, CA) with horseradish peroxidase detection and 3,3′,5,5′-tetramethylbenzidine (TMB, Fitzgerald Industries, Concord, MA) as the substrate as previously described^21^.

CYP450 enzyme activities were measured by first incubating cultures in substrates for 1 hour at 37 °C and then detecting either the luminescence or fluorescence of metabolites using previously described protocols^23^. In particular, CYP1A2 was measured by cleavage of ethoxyresorufin into fluorescent resorufin (Sigma-Aldrich, St. Louis, MO); CYP2A6 was measured by modification of coumarin to fluorescent 7-hydroxycoumarin (Sigma-Aldrich); and, CYP3A4 was measured by cleavage of luciferin-IPA into luminescent luciferin (Promega, Madison, WI).

In order to assess glucose output in MPCC supernatants, hypo-, normo- or hyperglycemic cultures were first incubated in hormone-free PHH culture medium for 24 hours, washed 3 times with 1X phosphate buffered saline (PBS, Corning Life Sciences), and then incubated for 4 to 8 hours in glucose-free and serum-free culture medium containing +/− gluconeogenic substrates (2 mM pyruvate from Corning and 20 mM L-lactate from Sigma-Aldrich) and +/− recombinant human insulin (1 to 100 nM, Sigma-Aldrich). Finally, glucose levels in collected cell culture supernatants were measured using the Amplex Red glucose/glucose-oxidase assay kit (Thermo Fisher Scientific).

### Cell staining

Cultures were fixed with 4% (vol/vol) paraformaldehyde (Alfa Aesar, Ward Hill, MA) for 15 minutes at room temperature (RT), washed three times with 1X PBS, and nuclei and intracellular lipids were stained. Briefly, cultures were incubated in a 300 nM 4′,6-diamidino-2-phenylindole (DAPI, MP Biomedicals, Solon, OH) solution for 10 minutes at RT, followed by incubation in a 10 μM Nile Red (AAT Bioquest, Sunnyvale, CA) solution for 10 minutes at RT. Cultures were then washed 3 times with 1X PBS and imaged using a DAPI light cube (excitation/emission: 357/447 nm) and GFP (green fluorescent protein) light cube (excitation/emission: 470/510 nm) on an EVOS FL Imaging System (Thermo Fisher Scientific). Nile red staining was quantified by measuring the pixel intensity per island of PHHs using the Image J software^52^.

FOXO1 immunofluorescent staining was carried out on normo- or hyperglycemic cultures after they had first been treated with hormone-free, serum-containing, 5 mM glucose medium for 24 hours and then treated with serum-free, glucose-free medium with +/− various concentrations of recombinant human insulin as described above. Cultures were fixed 1 hour after adding the aforementioned medium using 4% PFA in 1X PBS. Fixed cultures were first blocked for 1 hour at RT with blocking solution containing 5% (vol/vol) goat serum (Thermo Fisher Scientific) and 0.3% (vol/vol) Triton-X 100 in 1X PBS. Next, rabbit anti-human FOXO1 antibody (Thermo Fisher Scientific) was diluted in blocking solution at a concentration of 4 μg/mL and incubated with cells overnight at 4 °C. Cells were washed three times with 1X PBS, and then a secondary goat anti-rabbit IgG Alexa Fluor™ 555 conjugated antibody (Thermo Fisher Scientific) was added at a concentration of 20 μg/ml diluted in blocking buffer for 2 hours at RT. Cultures were then washed three times with 1X PBS, nuclei were counter-stained with DAPI, and cells were imaged using the EVOS FL Imaging System as described above. N:C ratios of FOXO1 labeling were obtained using image J software.

Viability staining of fibroblasts and MPCCs were carried out using ReadyProbes^®^ Cell Viability Imaging Kit (Thermo Fisher Scientific) and the EVOS FL Imaging System according to manufacturer’s protocol. Briefly, 2 drops of live cell, NucBlue®, reagent (Hoechst 33342) and dead cell, propidium iodide, reagent were added per mL of cell culture medium needed and then incubated with cultures for 15 minutes at 37 °C. Cell viability was assessed via the incorporation of propidium iodide into the nucleus and imaged using the EVOS FL Imaging System. Propidium iodide only incorporates into cells with a compromised cell membrane, while Hoechst 33342 can permeate the membranes of all cells and bind to the DNA in the nucleus.

Glycogen was visualized in fixed cells using the periodic acid-Schiff (PAS) kit (Sigma Aldrich) according to manufacturer’s recommendations. Briefly, fixed cultures were incubated with periodic acid solution for 5 minutes at RT. Cells were then washed 3 times with dH_2_O and Schiff’s reagent was added for 15 minutes at RT. Cells were then rinsed under tap water for 5 minutes continuously. Cultures were imaged with bright field microscopy and a color camera using the EVOS XL Core Cell Imaging System.

### Statistical analysis

Each experiment was carried out in duplicate or triplicate wells for each condition. Three cryopreserved PHH donors were used when available to confirm trends. Error bars represent standard deviation (SD). Microsoft Excel and GraphPad Prism 5.0 (La Jolla, CA) were used for data analysis and plotting data. Statistical significance of the data was determined using the Student’s *t*-test or one-way ANOVA with Dunnett’s multiple comparison test for post hoc analysis.

## Additional Information

**How to cite this article**: Davidson, M. D. *et al*. Long-term exposure to abnormal glucose levels alters drug metabolism pathways and insulin sensitivity in primary human hepatocytes. *Sci. Rep*. **6**, 28178; doi: 10.1038/srep28178 (2016).

## Supplementary Material

Supplementary Information
