# Supplementary material for: Long-term exposure to abnormal glucose levels alters drug metabolism pathways and insulin sensitivity in primary human hepatocytes
Source: Sci Rep. 2016 Jun 17;6:28178. doi: 10.1038/srep28178 (PMC4911593; doi:10.1038/srep28178)
Supplement: Supplementary Information [file srep28178-s1.pdf]

SUPPLEMENTAL INFORMATION

Long-term exposure to abnormal glucose levels alters drug metabolism pathways and insulin sensitivity in primary human hepatocytes

Matthew D. Davidson, Kimberly R. Ballinger, and Salman R. Khetani, PhD

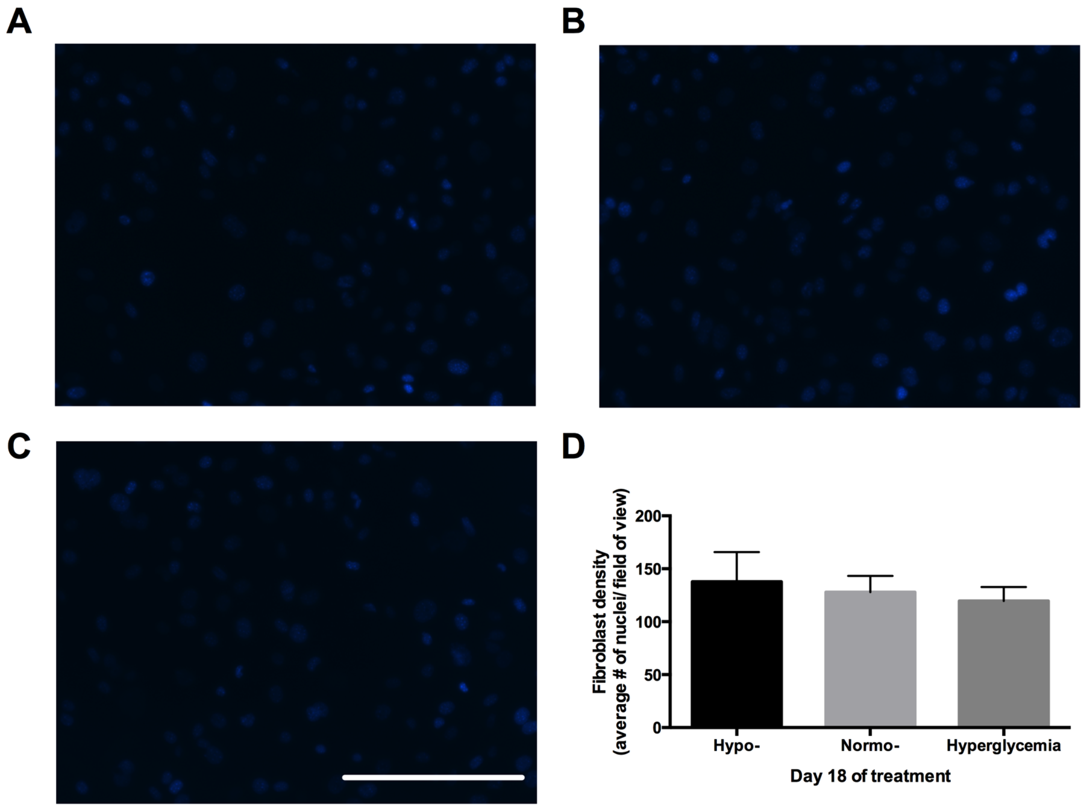

Supplemental Figure 1. Effects of glucose concentration on fibroblast numbers in MPCCs. MPCCs were fixed after 18 days of treatment with various glucose concentrations and stained with DAPI for cell counting. Images of 3T3-J2 fibroblasts between hepatocyte islands cultured in hypo- (0.4- 0.5 mM) (**A**), normo- (5 mM) (**B**) and hyperglycemic (25 mM) (**C**) culture medium on day 18 of treatment. Scale bar is 200 µm. (**D)** Average number of cells per field of view quantified using ImageJ software. Error bars represent SD.

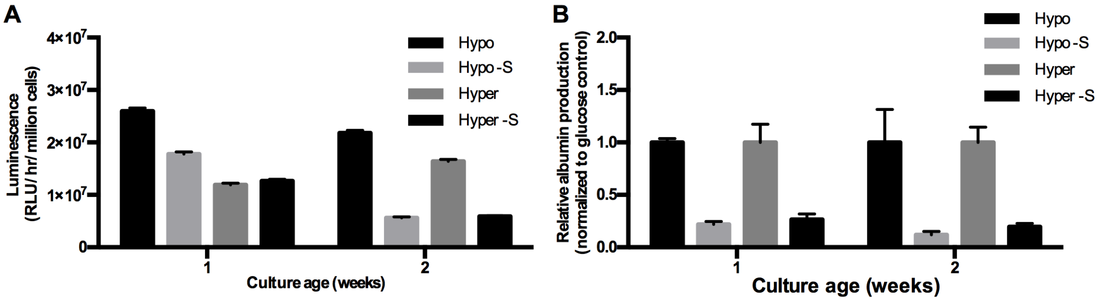

Supplemental Figure 2. Serum removal decreases hepatocyte functions in MPCCs. MPCCs were established for 4 days and then serum was removed from culture media. **(A)** CYP3A4 activity measured with Luciferin-IPA in hypo- and hyperglycemic medium with or without 10% (vol/vol) serum (-S). (**B)** Relative albumin production in hypo- and hyperglycemic medium with or without serum (-S). Data is normalized to the serum-containing control for each glucose level. Error bars represent SD across 3 wells. Similar results were observed in another primary hepatocyte donor.

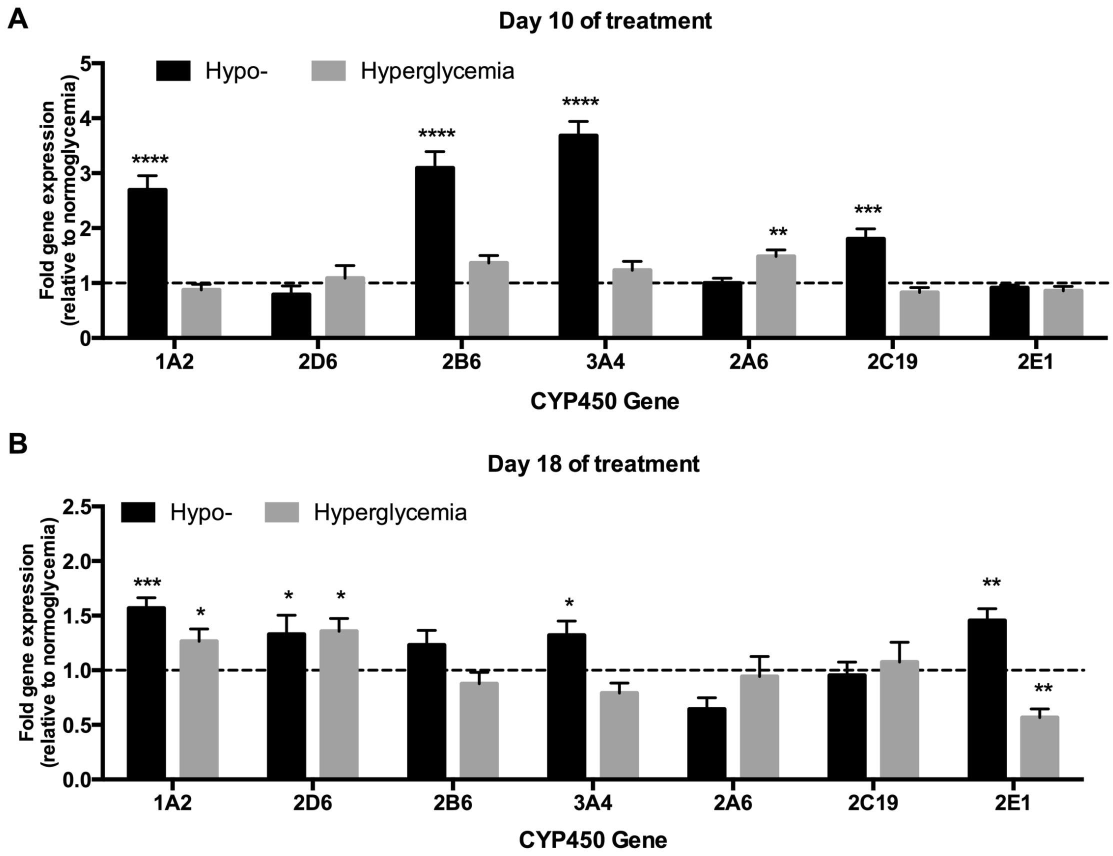

Supplemental Figure 3. Glucose-induced modulation of CYP450 gene expression in MPCCs. Levels of CYP1A2 *(1A2),* CYP2D6 (*2D6*), CYP2B6 (*2B6*), CYP3A4 *(3A4)*, CYP2A6 (*2A6*), CYP2C19 (*2C19*), and CYP2E1 (*2E1*) mRNA transcripts in MPCCs treated with hypo- and hyperglycemic culture media for 10 days (**A**) and 18 days (**B**). Data is normalized to the normoglycemic control. Error bars represent SD. * p≤ 0.05, ** p≤ 0.01, *** p≤0.001, and **** p≤0.0001.

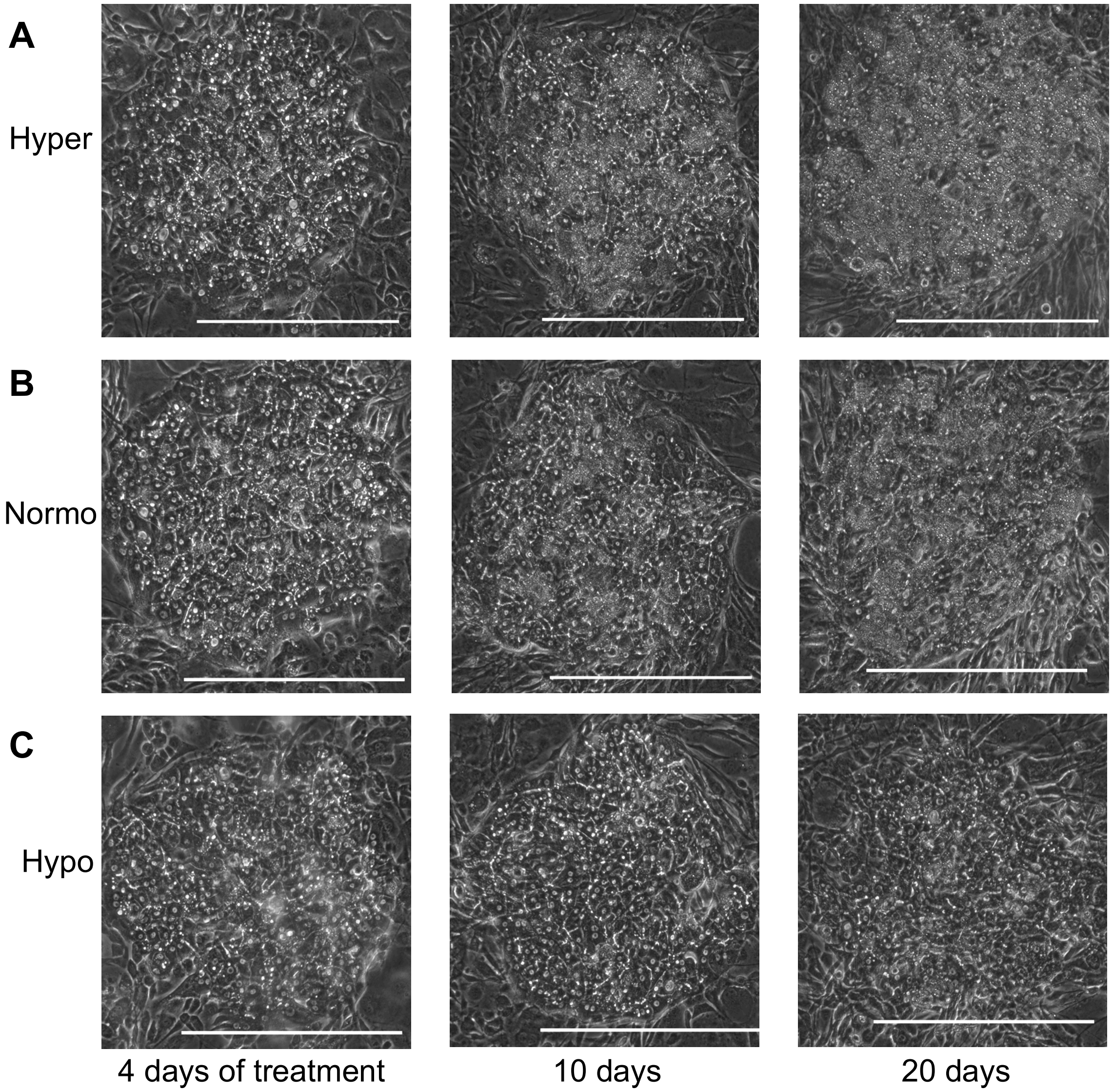

Supplemental Figure 4. Glucose-induced changes in hepatocyte morphology in MPCCs. Phase contrast image of PHHs in MPCCs treated over time with a hyper- (**A**), normo- (**B**), and hypoglycemic (**C**) culture medium. Scale bars represent 400 µm.

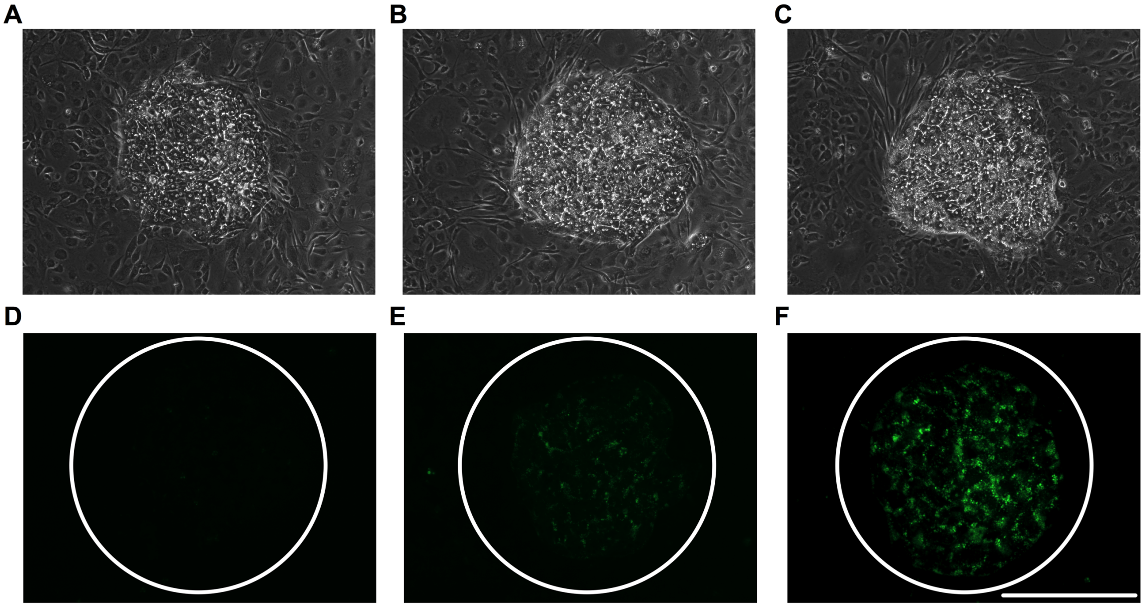

Supplemental Figure 5. Glucose-induced accumulation of neutral lipids in MPCCs after 10 days of treatment. Phase contrast images of MPCCs in hypo- (**A**), normo- (**B**) and hyperglycemic (**C**) culture medium after 10 days of treatment. Nile red (neutral lipids) staining of MPCCs in hypo- (**D**), normo- (**E**) and hyperglycemic (**F**) culture medium after 10 days of treatment. Circles highlight hepatocyte island location and scale bar is 400 μm.

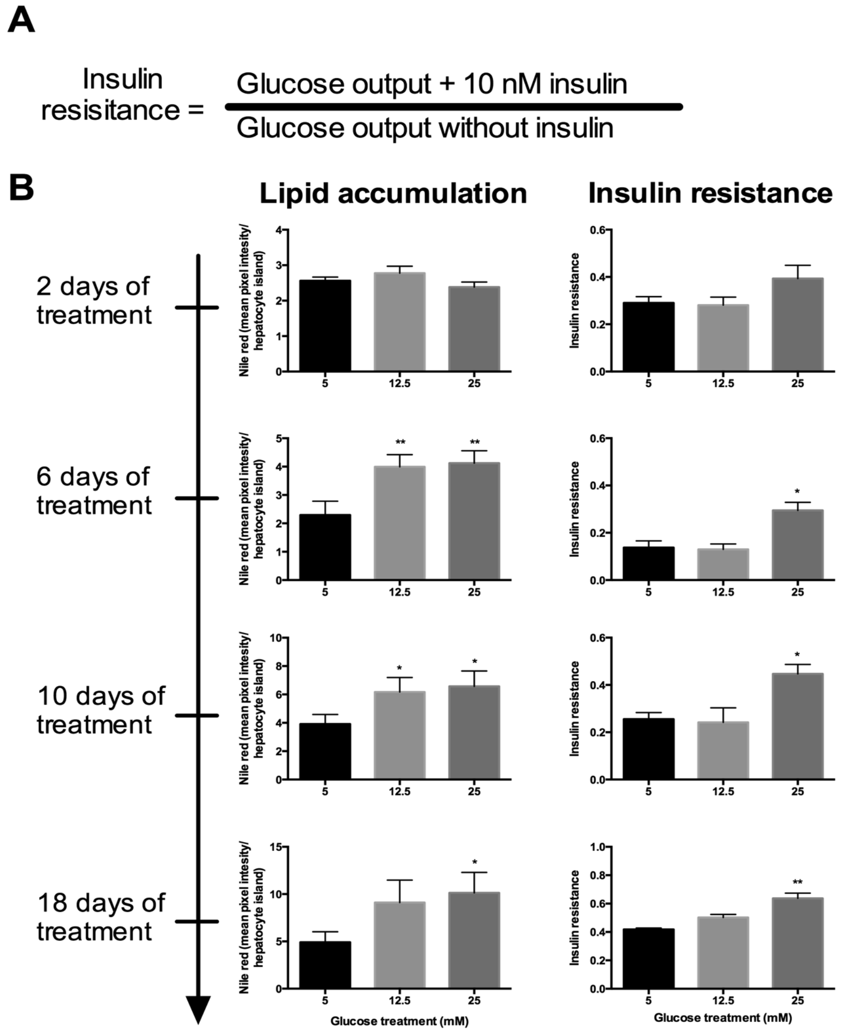

Supplemental Figure 6. Lipid accumulation and insulin resistance in MPCCs treated with varying glucose levels. (**A**) Insulin resistance is calculated by dividing the MPCC glucose output under insulin stimulation (10 nM) by the glucose output without insulin ("0" means complete inhibition of glucose output by insulin, while "1" means no effect of insulin on glucose output). (**B**) Time course of lipid accumulation, assessed via Nile Red fluorescence quantification (9 hepatocyte islands quantified per treatment), in MPCCs continuously treated with culture media containing 5 mM (normoglycemic), 12.5 mM or 25 mM (hyperglycemic) glucose. (**C**) Insulin resistance development over time in MPCCs treated as in panel (**B**). Error bars represent SD. * $p \leq 0.05$, and ** $p \leq 0.01$.

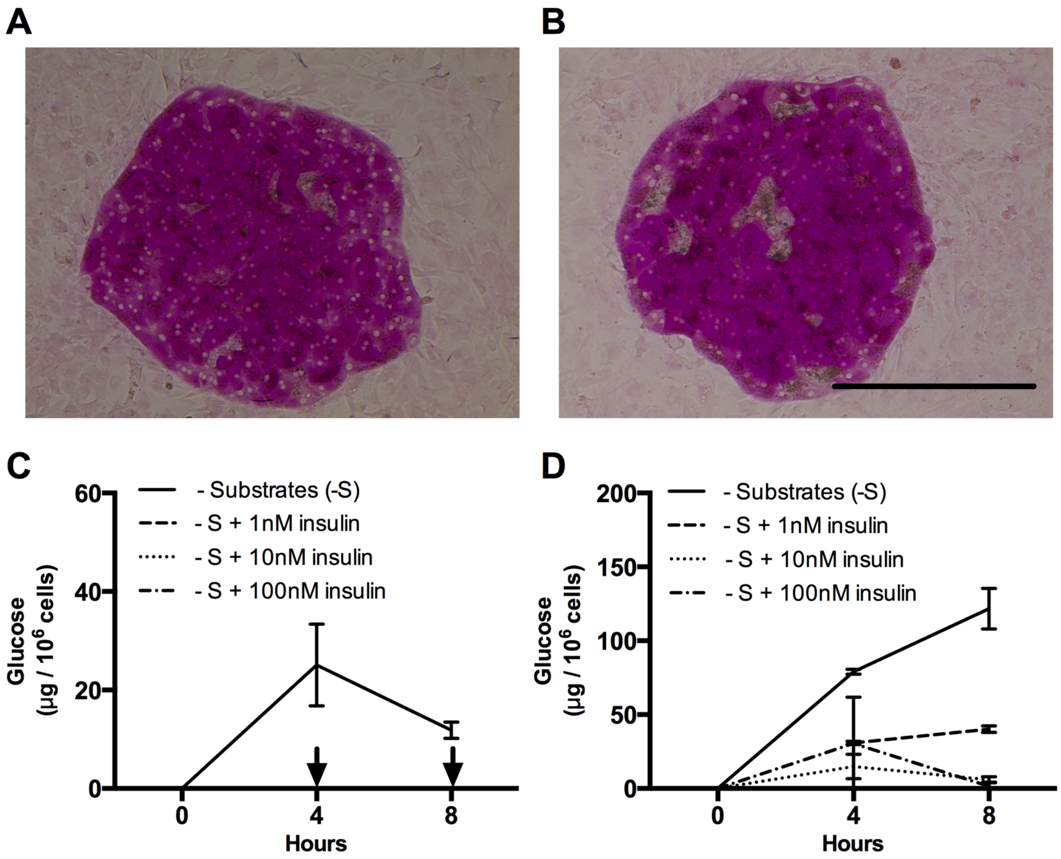

Supplemental Figure 7. Glucose-induced modulation of insulin sensitivity in MPCCs. PAS (glycogen) staining of MPCCs treated with either normo- (**A**) or hyperglycemic (**B**) culture medium for 10 days *prior* to assessing glucose output in the supernatants as in the subsequent panels of this figure. Scale bar is 400 µm. (**C**) Glucose output over time in supernatants of normoglycemic MPCCs (10 days of treatment) treated *without* any gluconeogenic substrates in the presence or absence of different levels of insulin (see methods for additional details). Arrows indicate no detectable glucose in supernatants. (**D**) Glucose output as in panel (**C**) except hyperglycemic MPCCs were used. Error bars represent SD across 3 wells. Similar results were observed in another primary hepatocyte donor.

SUPPLEMENTAL TABLE 1: Sequences for the GE Healthcare Dharmacon Solaris™ brand primer/probe sets used in this study.

**Gene: GAPDH**
Forward Primer: GCCTCAAGATCATCAGCAATG
Reverse Primer: CTTCCACGATACCAAAGTTGTC
Probe: GCCAAGGTCATCCATGA
**Gene: ARG1**
Forward Primer: ACTTGCATGGACAACCTGT
Reverse Primer: ATCCTGGCACATCGGGAAT
Probe: GAACTAAAAGGAAAGATTC
**Gene: ALB**
Forward Primer: AATGTTGCCAAGCTGCTGA
Reverse Primer: TCATCCCGAACTTCATC
Probe: CTGTTGCCAAAGCTCGATG
**Gene: AHR**
Forward Primer: CGTCTAAGGTGTCTGCTGGATA
Reverse Primer: CCCTTGGAAATTCATTGC
Probe: TCATCTGGTTTTCTGGCAA
**Gene: CYP3A4**
Forward Primer: CCTCATCCCAATTCTTGAAG
Reverse Primer: GCTGAAGGAAATCCACTC
Probe: GAAGATACACAAAAGCACCG
**Gene: CYP2A6**
Forward Primer: GATGACCACGTTGAACCT
Reverse Primer: ATGGACCTTGGCCTCCAC

Probe: ATGAAGCACCCAGAGGT
**Gene: MLXIPL**
Forward Primer: GTTTGATGACTACGTCCGAAC
Reverse Primer: GCCGGATGAGGATGCTG
Probe: AAGTTCTGGGTGTTCAG
**Gene: HMOX1**
Forward Primer: ATTGCCAGTGCCACCAAGTTC
Reverse Primer: CTCCTCAAAGAGCTGGATGT
Probe: AGACTGCGTTCCTGCTCAA
**Gene: G6PC**
Forward Primer: CCTACAGATTTCGGTGCTTG
Reverse Primer: TTCGTGACAGACAGACATTCAG
Probe: TTCTGGGCTGTGCAGCT
**Gene: PCK1**
Forward Primer: TCGAGAATAACGCTGAGCTGTG
Reverse Primer: TGAGAGCCAACCAGCAGTT
Probe: CTGAAGAAGTATGACAAC
**Gene: SLC2A2**
Forward Primer: GCTCAACTAATCACCATGCTCTG
Reverse Primer: GCTACTAACATGGCTTTGATTCTTC
Probe: CTTGGGGACACACTTGG
